# Public perceptions of predictive testing for rheumatoid arthritis compared to breast cancer and early-onset Alzheimer’s disease: a qualitative study

**DOI:** 10.1186/s41927-021-00244-w

**Published:** 2022-03-02

**Authors:** Juhi Singhal, Imogen Wells, Gwenda Simons, Sabine Wöhlke, Karim Raza, Marie Falahee

**Affiliations:** 1 College of Medical and Dental Sciences, University of Birmingham, Birmingham, UK; 2 Rheumatology Research Group, Institute of Inflammation and Ageing, College of Medical and Dental Sciences, University of Birmingham Research Laboratories, Queen Elizabeth Hospital, Birmingham, B15 2WB UK; 3 Department of Health Sciences, Faculty of Life Sciences, Hamburg University of Applied Sciences, Hamburg, Germany; 4 MRC Versus Arthritis Centre for Musculoskeletal Ageing Research, Birmingham, UK; 5 Research Into Inflammatory Arthritis Centre Versus Arthritis, College of Medical and Dental Sciences, University of Birmingham, Birmingham, B15 2TT UK

**Keywords:** Predictive testing, Risk perception, Rheumatoid arthritis, Breast cancer, Early-onset Alzheimer’s disease, Focus groups

## Abstract

**Background:**

There is increasing research focus on prediction and prevention of rheumatoid arthritis (RA). Information about risk of RA is increasingly available via direct-to-consumer testing. However, there is limited understanding of public perceptions around predictive testing for RA. This study explores public perceptions of predictive testing for RA in comparison to breast cancer (BC) and early-onset Alzheimer’s disease (AD).

**Methods:**

Four focus groups with 21 members of the public were conducted using hypothetical vignettes about predictive testing for each disease. Transcripts of focus group proceedings were analysed inductively using thematic analysis.

**Results:**

Thematic analysis of the data produced three key themes: decision-making factors, consequences, and consumer needs. Factors suggested that might influence decision-making about predictive testing included family history, fear, and perceived severity and treatability of the illness. RA was perceived to be less severe and more treatable than BC/AD. Potential consequences of predictive testing across all diseases included lifestyle modification, planning for the future and discrimination by employers or insurers. Predictive testing for RA was perceived to have less potential for negative psychological consequences than other diseases. Participants highlighted that individuals undertaking predictive testing should be signposted to appropriate support services and receive information on the accuracy of predictive testing. It was suggested that strategies to mitigate concerns regarding communication and confidentiality of risk results are required.

**Conclusions:**

The findings of this study reflect public misunderstandings regarding RA that may impact the uptake of and responses to predictive testing, and key informational needs of individuals considering a predictive test. Predictive strategies should be accompanied by awareness-raising initiatives and informational resources.

**Supplementary Information:**

The online version contains supplementary material available at 10.1186/s41927-021-00244-w.

## Introduction

Rheumatoid arthritis (RA) is a chronic inflammatory joint disease affecting 1 in 100 individuals and is characterised by pain, stiffness and swelling of joints [1–3]. Extra-articular symptoms include fatigue, depression and cardiovascular disease. The aetiology of RA is not fully understood, but several risk factors have been identified including HLA-DRB1 allele expression, a positive family history, smoking, obesity, and poor dental health [4]. Many patients have RA-related autoantibodies, notably rheumatoid factor and anti-cyclic citrullinated peptide antibody, which can be present for several years before the onset of symptoms [5–7]. Early diagnosis and treatment initiation improves clinical outcomes by slowing disease progression and increasing the chance of disease-modifying anti-rheumatic drug-free remission [8, 9]. There is currently no cure for RA, and despite therapeutic interventions, a proportion of patients will still develop irreversible joint damage and loss of function. RA presents significant personal and economic burden due to increased healthcare use, disability and reduced ability to work [10–12].

Pre-clinical phases of RA represent windows within which it may be possible to identify individuals at-risk of developing the disease [7]. Research efforts are being increasingly directed towards the accurate identification of individuals at-risk of RA to facilitate preventive interventions. As the development of predictive algorithms continues and prevention trials are ongoing, it is increasingly likely that disease risk estimates for RA based on clinical variables, genetics, and other biomarkers will become available to members of the public via healthcare professionals or commercial providers [13, 14]. Therefore, it is important to understand public opinion and needs around predictive testing for RA, to inform policy and regulation in this context. Stakeholder perceptions of predictive testing for RA will inform future development of tailored information resources and preventive strategies.

Previous qualitative research has explored the perspectives of patients with RA, first-degree relatives and seropositive individuals (with and without clinically suspect arthralgia) on predictive testing [14–19]. Findings from these studies revealed concerns regarding the probabilistic nature of the risk information and potential negative psychosocial impacts. These studies emphasised a need for better psychological support, effective communication of risk information and adequate provision of information in order to facilitate decision-making about whether to undertake predictive testing. In addition, a lack of understanding about RA and its associated risk factors was noted. Studies on stakeholder perceptions about predictive testing for other diseases have highlighted social and ethical issues associated with risk information [20–22].

Comparisons across disease areas can provide valuable insights into perceptual variability and information needs [20]. No studies to date have explored the perceptions of members of the general public about predictive testing for RA, or how these may relate to testing for other diseases. The present study explores the differences and similarities in attitudes towards predictive testing for RA with those for breast cancer (BC) and early-onset Alzheimer’s disease (AD). These diseases differ in terms of the availability of treatments, and public perceptions about their severity and controllability [20]. The perceptions of the public may identify additional ethical concerns and barriers to effective communication of risk status. The findings will facilitate future clinical implementation of predictive tools for RA that incorporate information about genetic and other biomarkers, by providing insight into the educational and psychological support needs of individuals considering predictive testing.

## Materials and methods

### Study design

This study was a qualitative study, with an inductive approach, using focus groups following a structured discussion guide incorporating case vignettes [23]. Focus group with members of the general public were conducted at the Medical School of the University of Birmingham, United Kingdom (UK) between September and October 2017, as part of an international multi-centre comparative study of public perceptions of recent developments in genetic testing [24, 25]. Ethical approval was granted by the University of Birmingham Science, Technology, Engineering and Mathematics Ethical Review Committee (ERN_17-0298). Study reporting follows the Consolidated Criteria for Reporting Qualitative Research (COREQ) guidelines [26]. The completed COREQ checklist is provided in Additional file 1. The focus groups were facilitated by MF and GS (both female academic psychologists (PhDs) with expertise in mixed methods and behavioural rheumatology). The discussions were led by MF, whilst GS took field notes and provided practical support. One focus group was additionally attended and observed by IW, a female postgraduate doctoral research student with a background in health psychology, for training purposes.

### Recruitment

Eligible participants were 18 years of age or older, able to attend a focus group at the University of Birmingham, and able to participate in a focus group conducted in English. Participants were recruited between June and October 2017. Recruitment was facilitated by posters distributed via a web-based recruitment platform for members of the public and social media networks. The study was additionally advertised via electronic mailing lists and virtual bulletin boards associated with the University of Birmingham. Those interested in taking part were asked to contact the researchers to register interest for further information via telephone or email. As participants were not approached by researchers we are unable to describe the characteristics or reasons of potential participants who chose not to participate. Potential participants were provided with a Participant Information Sheet and a summary of topics to be discussed during the focus groups, in advance of their participation in the focus groups. However, no information about the disease areas to be discussed was provided in advance of the focus groups. Individuals were contacted via phone or email to arrange participation in a focus groups. Participants were offered an incentive of £15 shopping vouchers in return for participating in the focus group.

### Data collection

Participants were advised they could withdraw from the study at any time without consequence or providing a reason. Written informed consent was obtained from all participants prior to taking part in the focus group. Participants were not previously known to the researchers, and researchers introduced their role, experience, and interest in the research topic to participants at the start of each focus group. Each participant was assigned an identification number. Participants were identified by number, and not by name on all study documents, including focus group transcripts. Participants completed a background questionnaire gathering sociodemographic information.

Focus groups were guided by a semi-structured interview schedule shown in Additional file 2. Participants were asked to discuss three hypothetical vignettes describing situations where an individual considers undertaking predictive testing for BC, early-onset AD and RA respectively. The order of presentation of the vignettes was fixed across the focus groups. After each vignette was introduced, PowerPoint slides were used to present hypothetical results of the predictive tests to encourage further discussion (Additional file 2). Risk results were presented as natural frequencies (e.g. 20 out of 100 people) and using icon arrays. Development of the vignettes for BC and early-onset AD is described in detail elsewhere [24, 25]. The vignette relating to RA was developed with input from KR, a male Professor of Rheumatology and Honorary Consultant Rheumatologist with expertise in risk assessment for RA [27, 28].

Participants were informed that there is currently no cure for RA, though for many people the symptoms can be managed with long-term immunomodulatory medication, especially if diagnosed at an early stage. After initial discussion about the value of predictive testing for RA, participants were asked to imagine that a person receives test results that reveal their risk of developing RA before the age of 55 is 20%. Participants were then prompted to discuss how this result would affect the recipient’s wellbeing and lifestyle, and who, if anyone the recipient should share the result with. Participants were asked to compare their responses for the RA vignette with those for the preceding vignettes relating to BC and early-onset AD. Following each focus group, the facilitators reviewed the main themes discussed, practical considerations and, where appropriate, whether thematic saturation had been achieved.

### Data analysis

The focus groups were audio-recorded and transcribed verbatim using an independent transcription company. The objectives of the analysis were to explore perceptions of predictive testing for RA, and compare them with those for BC and early-onset AD. Analysis of the transcripts was conducted following an inductive thematic approach, facilitated by the NVivo software (version 12.0) and supplemented with researchers field notes [29]. The data were coded independently by JS and IW. Continuous comparison of the codes and development of themes was undertaken by discussion within the entire research team until there was consensus that thematic saturation had been reached.

## Results

### Participant characteristics

Four focus groups were conducted with a total of 21 participants, including 13 females (61.9%). The majority of the participants were aged between 18 and 35 (61.9%) and had no children (66.7%). Participants were ethnically diverse with six individuals identifying as Asian (28.6%) and three individuals identifying as Black or African-Caribbean (14.3%). 11 participants (52.4%) were employed either full-time or part-time, and nine participants (42.9%) attained a degree-level of education. Some participants had prior experience of genetic testing (28.6%) and genetic screening (4.8%). The characteristics of the participants are presented in Table 1. The durations of the focus groups were between 90 to 120 min, and group size varied between four to seven participants.

**Table 1:** Participant characteristics

Participant ID number	Gender	Age	Marital status	Children	Employment status	Highest level of education	Ethnicity	Experience with GT or GS
1	Male	51–70	Married	Yes	Self-employed	Degree level	Other Asian	No
2	Female	51–70	Single	No	Employed	Postgraduate qualification	White British	Yes (GT only)
3	Female	51–70	Married	Yes	Employed	GCSE/O level	White British	Yes (GT only)
4	Male	26–35	Single	No	Employed	Degree level	Asian Pakistani	No
5	Male	71+	Married	Yes	Self-employed	Postgraduate qualification	White British	Yes
6	Female	51–70	Married	Yes	Retired	GCSE/O level	White British	No
7	Female	26–35	Single	No	Student	Degree level	Black Caribbean	No
8	Male	26–35	Single	No	Employed	Degree level	White British	No
9	Female	26–35	Single	Yes	Employed	Postgraduate qualification	White British	No
10	Male	51–70	Married	Yes	Unemployed	Vocational qualification	Asian Indian	No
11	Female	18–25	Single	No	Student	Degree level	White British	Yes (GT only)
13	Female	26–35	Single	No	Employed	Degree level	White British	Yes (GT only)
15	Female	26–35	Single	No	Employed	Postgraduate qualification	Asian Indian	No
17	Male	51–70	Single	No	Employed	Degree level	White British	No
18	Male	18–25	Partnership	No	Employed	A-level	White British	No
19	Female	18–25	Single	No	Employed, Student	A-level	Asian Chinese	No
20	Female	18–25	Partnership	No	Employed, Student	Degree level	Mixed Caribbean	No
21	Female	26–35	Single	No	Student	Degree level	N/A	No
22	Female	26–35	Single	No	Student	Postgraduate qualification	Other White	No
23	Female	18–25	Single	No	Student	A-level	Black African	No
24	Male	71+	Married	Yes	Retired	Postgraduate qualification	Mixed Asian	Yes (Both)

*GT* genetic testing, *GS* genetic screening

### Codes and themes

Three key themes were identified: decision-making factors, consequences and consumer needs. The key themes and subthemes are summarized in Table 2 and described below using supporting quotations, referred to in the text using ‘Q’ followed by the code number. The supporting quotations are presented in Tables 3, 4, and 5.

**Table 2:** Overview of themes and sub-themes

Theme	Subtheme	Diseases discussed*
Decision-making factors influencing uptake of predictive testing	Perceived severity of disease	RA BC AD
	Fear of being identified as at high risk	BC AD
	Treatability of disease	RA BC AD
	Family history	RA BC AD
	Family structure	BC AD
	Age	RA BC AD
	Health attitudes	RA BC
	Upbringing	RA BC AD
	Comorbidities	BC
	Research	RA BC AD
	Occupation	RA
	Existing screening services	BC
	Media	BC
Potential consequences of predictive testing	Lifestyle modification	RA BC AD
	Future planning	RA BC AD
	Responsibility to Disclose Risk Information	RA BC AD
	Risk of discrimination	RA BC AD
	Psychological impact	BC AD
	Support	BC
	Self-learning	RA
	Early diagnosis	BC
Information and support needs of consumers of predictive testing	Test accuracy	RA BC AD
	Risk management	RA BC
	Communication of risk information	RA BC
	Support services	BC

*RA rheumatoid arthritis, *BC* breast cancer, *AD* Alzheimer’s disease

**Table 3:** Quotations relating to decision-making factors influencing uptake of predictive testing

Code	Quotation	Disease
*Perceived severity*		
Q1	It’s such a huge thing, it’s not like I’m not saying cancer isn’t huge but like this is a lot more, there’s no going back from it. (Participant 19)	Early-onset AD
Q2	No one really wants to know a death sentence. (Participant 22)	Early-onset AD
Q3	You think cancer ‘oh I’m going to die’, Alzheimer’s ‘I’m getting old I’m gonna die’ but rheumatoid arthritis I just think ‘oh that’s pain’. (Participant 13)	Early-onset AD, BC, RA
Q4	The rheumatoid arthritis is not going to kill Miss Jones, so it’s not a life threatening disease. (Participant 1)	RA
Q5	I think it’s slow just achy joints, cranky knees, your hands are a little bit stiff and a few months pass before you think to go to the doctors. (Participant 3)	RA
Q6	It never even crossed my mind that I could go onto develop it, didn’t realise, lack of education there. (Participant 3)	RA
*Fear of being identified as at high risk*		
Q7	I don’t know if I’d want that hanging over me because it terrifies me, absolutely terrifies me. (Participant 8)	Early-onset AD
Q8	There is that side of being scared. (Participant 19)	Early-onset AD
Q9	A lot of people would shy away from it because of the fear. (Participant 8)	BC
*Treatability*		
Q10	I think I would go less likely to have a test, if I knew it was a degenerative condition which had no treatment. (Participant 2)	Early-onset AD
Q11	I’d be less inclined to take it because at least with the breast cancer as you say, it’s treatable, if you find out there’s some benefit to it. (Participant 18)	Early-onset AD, BC
Q12	I would only take the test if I knew that the result, I could change it based on my lifestyle, I’d be much more likely to get it I think. (Participant 20)	RA
*Family history*		
Q13	If I know I’ve got the family history I’ve probably grown up expecting it. (Participant 2)	Early-onset AD
Q14	Knowing I had a family history would be good enough for me, I wouldn’t want to know too much more after that. (Participant 23)	Early-onset AD
Q15	If you kind of know you have a family history you might be already, not necessarily taking precautions but doing stuff like for that…I probably wouldn’t take the test because being myself I probably would have searched it up already. (Participant 23)	RA
Q16	I would want to know and we’ve had quite a lot of experience with Alzheimer’s with friends and family. (Participant 3)	Early-onset AD
Q17	If there has been a positive outcome in the family, then she would be very much inclined to do so, but if there has been negativity towards it, then she might consider not doing it. (Participant 1)	BC
Q18	If they’ve had a family history as well, they’ve seen members of their family go through it and they want to prevent that happening. (Participant 9)	BC
*Family structure*		
Q19	What is her domestic situation? Is she on her own, is she as you pointed out looking after somebody as a carer? (Participant 5)	Early-onset AD
Q20	I think it would depend on whether or not she has kids or if she’s planning to have kids. (Participant 15)	Early-onset AD
Q21	I think most women, particularly if they’ve got children would want to have the test. (Participant 3)	BC
Q22	If she’s got daughters she might want to find out in case her daughters might develop breast cancer in the future. (Participant 6)	BC
*Age*		
Q23	I’d think ‘well maybe I can wait until that age’, I’m only this age now so I don’t need to know. (Participant 9)	RA
Q24	A young person may not be thinking that far ahead anyway. (Participant 17)	BC
Q25	It’s not until you hit perhaps 40 and you seem to become aware of various illnesses and the frailty of life…I don’t know whether a youngster would want to go down the route of finding out (Participant 3)	Early-onset AD
*Health attitudes*		
Q26	I think some people want to do everything they personally can to reduce and modify their risks, and other people say ‘well if it’s going to happen, it’s going to happen’. (Participant 2)	RA
Q27	A lot of people wouldn’t do it because they don’t want to change who they are, they don’t want to change their lifestyle. (Participant 11)	BC
*Upbringing*		
Q28	The level of the person’s education and knowledge and experiences, that would have an impact on the decisions they make. (Participant 4)	RA
Q29	Every individual is different and dependent on what her upbringing is and the choices she makes then it would have an impact. (Participant 4)	BC
Q30	It’s about your mentality and the way you think about things (Participant 21)	Early-onset AD
*Co-morbidities*		
Q31	Has she got any other illnesses. (Participant 5)	BC
*Research*		
Q32	I think in the interest of science and progression it would probably motivate them to go and have it done so that they can help with research or in any way contribute towards that kind of research for the future. (Participant 1)	RA, Early-onset AD, BC
*Occupation*		
Q33	It could depend on what kind of job you do, so if it’s like an office type job or a physical one, and when the pension age is at the time. (Participant 17)	RA
*Existing screening services*		
Q34	We’re supposed to be checking for breast cancer anyway, so unless it would put her on a kind of special you know, some kind of special treatment that she would get. (Participant 22)	BC
Q35	I’ve got to this age so I’m eligible for the screening now, so I don’t need to have further testing. (Participant 9)	BC
*Media*		
Q36	I’m starting to think about Angelina Jolie now, so I don’t know, things also like media influence. (Participant 21)	BC

**Table 4:** Quotations relating to potential consequences of predictive testing

Code	Quotation	Disease
*Lifestyle modification*		
Q37	You could change your lifestyle, eat healthier, exercise, keep the brain active, those kind of stuff. (Participant 8)	Early-onset AD
Q38	If I find out I’ve got a particular genetic trait then will I be able to change my lifestyle and reduce that risk. (Participant 9)	BC
Q39	It gives you a chance to get yourself fit and reduce your risks like smoking, and they sometimes say pollution and all sorts of things. (Participant 3	RA
Q40	Perhaps presented in your own personal profile it might make you change your mind, it might take on a bit of extra significance. (Participant 20)	BC
Q41	If they don’t have this gene they could think I’m completely fine, I’m not going to get it. (Participant 19)	BC
Q42	I might stop doing all the good things like cycling and running and being healthy, I might stop doing all the preventative stuff because I thought the risk was remoter than I would have anticipated. (Participant 2)	RA
*Future planning*		
Q43	You can make allowance in terms of financial, putting money away for care and treatments that is you know that’s what you’re expecting later down the line. Make sure you’ve got a Power of Attorney. A good Will. (Participant 18)	Early-onset AD
Q44	If you know there’s a likelihood you’re going to develop it you can start to put things in place for the future and you know address your home and make it safe and start to do procedures…make sort of memory boards and that sort of thing. (Participant 3)	Early-onset AD
Q45	Expecting the worst you could put things in place so that you know if the worst were to happen that you know, things like your family were looked after and things like life insurance. (Participant 8)	BC
Q46	If you’ve got children and you need to prepare to leave them something to look after themselves, so your lifestyle might change in that you’re cutting back on doing things. (Participant 9)	BC
Q47	If you were going to buy a house and there’s a chance of you having rheumatoid arthritis, would you buy a house with stairs or get a bungalow. (Participant 10)	RA
Q48	If you’re a builder and the pension age is probably 89 by the time we get there, you’ll be thinking ‘oh god I can’t be a builder for the next 25 years’ you’d have to look at alternate career options. (Participant 17)	RA
Q49	I think it would like change your career path or your life. (Participant 11)	RA
*Responsibility to disclose risk information*		
Q50	I’m not sure I would rush to share it with my children. (Participant 2)	RA
Q51	She should probably let her kids know. (Participant 22)	Early-onset AD
Q52	As much as it is a personal choice, I feel like if that was me I’d feel a certain amount of responsibility to tell people I’m related to that I have a genetic predisposition. (Participant 11)	BC
Q53	If she has daughters she should certainly talk about it to them I think, so that they can be aware and perhaps take the test if necessary. (Participant 6)	BC
*Risk of discrimination*		
Q54	I’d be very careful about letting it get out into the public sphere because there is a lot, even when you don’t have insurance companies, but still if a company knows that you’re sick or have a higher risk of being sick, they’re not going to hire you. (Participant 22)	BC
Q55	I think possibly everyone in terms of jobs, work situations and things like that, how employers would be if that information was disclosed, you know employers would start questioning that person’s reliability… confidential disclosure would have to be carefully considered. (Participant 1)	Early-onset AD
Q56	If genetic testing became part of everyday life then the insurance company would certainly put a clause in there ‘have you had any genetic testing done?’ and that could impact on whether they insure you or not or how much you have to pay for insurance. (Participant 1)	Early-onset AD
Q57	We can say there is confidentiality it’s highly unlikely that information gets out, but that information is still there and it could get out, and that would impact your ability to work. (Participant 22)	RA
*Psychological impact*		
Q58	It’s always better to know either way because she’ll just spend her life worrying, but at least the test would give her an answer so to speak. (Participant 20)	BC
Q59	It might have a higher impact for your mental health. (Participant 2)	Early-onset AD
Q60	Tortured for a long time with whether she would or she wouldn’t get the diagnosis. (Participant 17)	Early-onset AD
Q61	Mentally about living with what could be a ticking time bomb, there are some I think who would find it very very difficult to carry on life as normal, it would forever be with them that they were at this increased risk. (Participant 2)	BC
Q62	It could also give her unnecessary worry. (Participant 22)	BC
*Support*		
Q63	I’d also look at support services around as well, because even though it isn’t a diagnosis, certain people might require additional support for you know, so as not to worry or you know drive themselves mad thinking about it constantly. (Participant 8)	BC
Q64	If they need someone to talk to about it…so maybe just also maybe even it it’s just a one-off session to talk to maybe a professional. (Participant 23)	BC
*Self-learning*		
Q65	It gives you the chance doesn’t it, to go out and educate yourself, find out what drugs are out there and what the scenario is going to be in the future. (Participant 3)	RA
Q66	It would make you more aware of educating yourself as to what to look for. (Participant 3)	RA
Q67	If you know there’s a likelihood of it developing, even if it’s only 20%, you would then perhaps think ‘oh my joints are a bit sore, I haven’t noticed that before’, it would make you do a bit of research and you found out about it, and you would actually be more aware of your body, the changes in your body. (Participant 3)	RA
*Early diagnosis*		
Q68	An earlier diagnosis will mean it’s more treatable. (Participant 19)	BC
Q69	It could make you more vigilant to do self-testing. (Participant 13)	BC
Q70	Being able to have access to more frequent mammograms and stuff like that. (Participant 23)	BC

**Table 5:** Quotations relating to information and support needs of consumers of predictive testing

Code	Quotation	Disease
*Accuracy*		
Q71	How thorough is the genetic testing, and the reliability of that…how much research is out there to prove that it’s effective in terms of for the individual? (Participant 4)	BC
Q72	The question that would come to my mind is how reliable is the testing…how accurate is this test. (Participant 4)	RA
Q73	I think I’d need to know about false positives and false negatives in that kind of percent [20% risk from genetic test]. (Participant 2)	RA
Q74	Going back to the actual testing, I mean if it’s as broad the result, currently with the technology that we have, how useful is that to know anyways. (Participant 17)	Early-onset AD
*Risk management*		
Q75	The first thing she would want to know is what are her options in terms of treatment, or all the options available to her. (Participant 1)	BC
Q76	They may want to know if there’s any preventative things they can do if they do find out they’re at risk (Participant 9)	BC
Q77	It would be useful to have ways in which you could potentially reduce your risk…if they didn’t have it then they might not necessarily go out on their own to do their own research. (Participant 7)	RA
Q78	Giving them the whole package when you are giving the results. (Participant 7)	RA
Q79	I would want to speak to experts before having a test; is this really going to make any difference if I know, and are there treatments I can have, or things that I can do that are preventative. (Participant 9)	RA
*Communication of risk information*		
Q80	It needs to be worded carefully not to scaremonger people. (Participant 7)	RA
Q81	If they’re given too much information, then they feel overwhelmed and not able to make a decision. The clinicians would have to find the balance on how much information they give the person having the test so that they can make an informed decision but not be sort of swayed by fear. (Participant 9)	BC
Q82	Need to balance the personal yet collective way of explaining things carefully. (Participant 7)	RA
Q83	I just think either some context to the numbers or just that it should be just explained exactly what these numbers mean rather than just spitting a number out, I don’t think it really explains the full breadth of the situation. (Participant 22)	BC
*Support services*		
Q84	Waiting for the results to come back and stuff like that is one of the worst times. (Participant 8)	BC
Q85	I’d also look at support services around as well, because even though it isn’t a diagnosis, certain people might require additional support for you know, so as not to worry or you know drive themselves mad thinking about it constantly. (Participant 8)	BC
Q86	There also needs to be a bit of care for the person who the test has been taken on…giving the person the result and just like ‘yeah you’re likelihood of getting breast cancer is 99%’ and they’ve got no-one to turn to, so I think it’s really important that the support network is there for the person. (Participant 7)	BC

#### Factors affecting decision-making about predictive testing

This key theme describes participants’ views on factors that influence an individual’s decision to engage in predictive testing, and how this varies according to disease context. Factors mentioned include perceived severity and treatability of the illness, family history, fear of a negative outcome, age, occupation, health attitudes, social upbringing, co-morbidities, existing health services and media. The supporting quotations are shown in Table 3.

Participants were more likely to have positive views about predictive testing if they perceived a disease as severe and life-threatening. Early-onset AD was perceived as most severe and was likened to a “death sentence” (Q1–2), whereas RA was regarded as the least severe and was not perceived to be life-threatening (Q3–4). The participants described RA as a disease of “just achy joints and cranky knees”(Q5). Conversely, participants stated that, compared with testing for RA, individuals may be less likely to undergo predictive testing for early-onset AD and BC, due to the fear of discovering they may be at high-risk of AD or BC. (Q7–9). Being identified as being at high risk of RA appeared to be less fear-provoking.

The participants’ views on predictive testing were influenced by the perceived treatability of a disease. Participants stated that they would be less likely to engage in predictive testing for early-onset AD compared to BC, since there is currently limited pharmacological treatment for this condition (Q10–11). Participants further stated that they would be more likely to engage in predictive testing for RA if preventive strategies were available (Q12).

Family history seemed to variably affect decisions regarding predictive testing. Participants suggested that individuals with a family history of early-onset AD or RA would be less likely to undertake predictive testing since they may already be aware of their increased susceptibility (Q13–15). However, participants also stated that witnessing the effect of BC or early-onset AD on a family member would motivate an individual to undertake predictive testing (Q16–18). Some participants suggested that individuals would be more likely to undergo predictive testing for early-onset AD and BC if they had dependents, particularly children (Q19–22). In contrast, being a parent or carer was not perceived to influence an individual’s decision to engage in predictive testing for RA.

Age was suggested to affect the decision to undertake predictive testing (Q23–25) for all of the diseases that were discussed, including RA. Participants stated that younger individuals may be less likely to engage in predictive testing across all diseases, since they may be less concerned about disease onset which was perceived to occur in the later stages of life. Health attitudes and behaviours were suggested to influence decision making regarding predictive testing across disease areas, particularly in relation to willingness to modify lifestyle to reduce disease risk (Q26–27).

Other factors perceived to influence the likelihood of an individual engaging in predictive testing for all disease areas were level of education and the presence of co-morbidities (Q28–30). It was suggested that facilitating research advancements in personalised medicine may additionally motivate individuals to undertake predictive testing (Q32). Participants noted that an individual’s occupation and career aspirations may affect their interest in predictive testing for RA (Q33).

External factors such as existing healthcare services and media were also perceived to influence the uptake of predictive testing for BC. Participants stated that individuals may be less likely to undergo predictive testing for BC since a screening programme already exists (Q34–35). It was suggested that interest in predictive testing for BC may be greater than for other conditions due to greater awareness raised by high-profile celebrities (Q36).

#### Potential consequences of predictive testing

This theme describes participants’ views on the potential consequences of engaging in predictive testing and how this may vary according to disease context. Potential consequences of predictive testing discussed included lifestyle modifications, advanced future planning, research advancements, psychological effects, responsibility to disclose risk information to family members, risk of discrimination, early diagnosis and opportunities for self-learning. The supporting quotations are shown in Table 4.

Participants stated that individuals may be more likely to address their lifestyle to reduce their risk of developing the disease as a result of predictive testing for each of the diseases studied; for example smoking cessation, eating a balanced diet and exercising (Q37–40) were mentioned. However, participants also noted that individuals identified as low-risk of developing RA and BC may feel falsely reassured and consequently stop practising healthy behaviours (Q41–42).

Predictive testing was perceived to facilitate advanced planning for the future. It was suggested that individuals found to be at-risk of early-onset AD or BC could prepare for incapacity or death by preparation of their will, care plans, financial savings (Q43–46). In contrast, risk results for RA were perceived to potentially influence an individual’s decisions about house purchases or career options (Q47–49).

The responsibility of individuals to disclose risk results to family members was perceived to be disease-dependent. Individuals undertaking predictive testing for RA were perceived to have little responsibility to share their risk results (Q50), whereas for BC and early-onset AD importance was often placed on individuals informing their children, especially to inform daughters about BC risk (Q51–53). Participants also raised concerns about the risk of discrimination by employers and insurance companies across all diseases, highlighting a need of reassurance regarding confidentiality for potential consumers (Q54–57).

Predictive testing for early-onset AD and BC was perceived to have potential psychological consequences, though this was not discussed in relation to RA. It was suggested that predictive testing for early-onset AD and BC could have a positive psychological impact health as it reduces uncertainty around disease development (Q58). Conversely, the potential for knowledge of risk status to cause considerable distress was also discussed (Q59–62). It was also suggested that individuals undertaking predictive testing may require additional support to cope with their risk results (Q63–64).

Participants noted that predictive testing for RA has the potential to raise personal awareness of symptoms and treatment options, (Q65–67). In contrast, predictive testing for BC was likely to be associated with earlier diagnosis and consequently better prognosis because at-risk individuals may be more likely to regularly self-check their breasts or access screening services (Q68–69). Participants further suggested that individuals established to be at risk of BC should be offered more frequent mammograms and access to surveillance (Q70).

#### Information and support needs for consumers of predictive testing

This theme describes the information and support needs that participants discussed in relation to predictive testing, and how these may vary according to disease context. The supporting quotations are shown in Table 5.

Participants stated that, irrespective of disease context, potential users of risk assessment interventions should be provided with information on the accuracy of such assessments and evidence in support of predictive testing across all diseases (Q71–74). Additionally, it was suggested that information on available treatment options and prevention strategies should be offered to potential consumers to enable informed decision-making on participation in predictive testing for RA and BC (Q75–79). This informational need was not discussed in relation to early-onset AD.

Participants further acknowledged that risk results across all diseases should be communicated in a manner that ensured understanding but avoided individuals feeling overwhelmed (Q80–83). Due to the likelihood of negative psychological consequences such as worry, it was perceived to be important that all individuals engaging in predictive testing were signposted to appropriate support (Q84–86). The support needs of individuals engaging in predictive testing were more often discussed in relation to BC than they were in relation to early-onset AD and RA.

## Discussion

### Summary of findings

This qualitative study explored public perceptions of predictive testing for RA in comparison to early-onset AD and BC in relation to three key themes: decision-making factors, consequences and consumer needs. In each of these areas, there were key differences in participants’ viewpoints according to the disease in question, which often related to the participants’ perceptions that RA was more treatable and had less of a negative impact on people’s lives than the other diseases discussed. This finding is in agreement with prior research describing commonly held misperceptions about RA [15, 16].

Factors suggested to influence an individual’s decision to engage in predictive testing across all diseases included perceived severity of the illness, treatability, family history, age, health attitudes, upbringing and the presence of co-morbidities. Results for BC and AD aligned with those of a similar study in Germany [25]. However predictive testing was more often perceived in a positive light for BC and AD than for RA in the present study.

Potential consequences of predictive testing across all diseases included implementation of healthy lifestyle modification and advanced future planning. Consistent with previous research on public perceptions of predictive testing in other disease contexts, the participants reported concerns relating to the risk of discrimination by employers and insurance companies [21]. The psychological impact of predictive testing for individuals was perceived to be much greater for early-onset AD and BC in comparison to RA. Furthermore, there was more ambiguity regarding an individual’s responsibility to disclose risk results for RA compared to both early-onset AD and BC. Predictive testing involving genetic information raises complex ethical issues around responsibility relating to the individual, their family, and their healthcare professionals, which are discussed extensively elsewhere [25, 30]. Our results highlight the importance of understanding the interplay between these ethical issues and illness perceptions, and how this interplay varies across diseases. It was suggested that predictive testing may motivate at-risk individuals to increase awareness of early symptoms and treatment options. This could have a positive impact on help seeking for RA, as delayed help seeking for RA is known to be a major source of treatment delay and worse clinical outcomes [31].

There were fewer differences across the diseases addressed in this study in relation to the information needs and support services that participants suggested were important for those considering/taking predictive tests. Information on the accuracy of predictive testing was perceived to be important in all disease contexts. In addition, the perceived educational needs of potential consumers of predictive testing for both RA and BC included information on available treatment options and preventive strategies. In accordance with previous research, the participants’ discussions highlighted a need for awareness-raising initiatives and educational resources centred on RA [14, 15]. Although the likelihood of negative psychological consequences in relation to predictive testing for RA was thought to be minimal, it was suggested that consumers of predictive testing across all disease contexts should be signposted to appropriate support services.

### Strengths and limitations

To the authors’ knowledge, this is the first qualitative study to explore public perceptions of predictive testing for RA in comparison with other disease areas. Our study sample is well- balanced across genders, ages and ethnicities. However, there are some limitations. Firstly, the sample was limited to individuals who were able to attend a focus group in-person in Birmingham, UK and may not be representative of other populations. Further work is needed to understand how perspectives on predictive testing vary across populations and cultures [32]. Secondly, it is possible that our sample is compromised by selection bias, since participants may have been more interested in health issues compared to the general population. Thirdly, the order of the disease vignettes was not alternated across the focus groups. Therefore, it is possible that discussion about early-onset AD and BC vignettes before that relating to RA influenced the responses provided by the participants to the third vignette. Finally, the participants responses to hypothetical vignettes may not reflect real world responses to predictive testing [33]. Whilst the current analysis generates insights by comparing RA with BC and AD, the findings may not transfer to comparisons with other diseases. Whilst this qualitative study generated rich insights about perceptions of predictive testing for RA by asking participants to consider comparisons between disease areas, it was not designed to test formal hypotheses about differences between diseases. Quantitative studies designed to address the latter objective would be an interesting area of further investigation.

Notwithstanding these potential limitations, this exploratory study demonstrates that perceptual variation across disease contexts is worthy of further investigation to inform policy and practice in this rapidly evolving area.

## Conclusion

In conclusion, there was considerable overlap across all three diseases regarding decision-making factors, consequences and information needs. However, predictive testing was perceived to be of less utility for RA, and to be associated with fewer psychological and moral consequences than BC or early-onset AD. The potential for predictive testing to result in earlier intervention was noted for RA and BC. The information and support needs identified in this study across disease areas should be incorporated into tailored resources to support informed decision-making and service design of future predictive testing programs. Strategies to mitigate concerns regarding communication and confidentiality of disease risk results are required.

## Supplementary Information

**Additional file 1.** COREQ checklist.

**Additional file 2.** Semi-structured interview schedule.

## Data Availability

The datasets used and/or analysed during the current study are available from the corresponding author on reasonable request.

## Abbreviations

RA: Rheumatoid arthritis
BC: Breast cancer
AD: Early-onset Alzheimer’s disease
UK: United Kingdom
COREQ: Consolidated criteria for reporting qualitative research guidelines
Q: Supporting quotations
