# Supplementary material for: Public perceptions of predictive testing for rheumatoid arthritis compared to breast cancer and early-onset Alzheimer’s disease: a qualitative study
Source: BMC Rheumatol. 2022 Mar 2;6:14. doi: 10.1186/s41927-021-00244-w (PMC8889636; doi:10.1186/s41927-021-00244-w)
Supplement: Supplementary file 2 — Additional file 2. Semi-structured interview schedule. [file 41927_2021_244_MOESM2_ESM.docx]

**Additional file 2:** Semi-Structured Interview Schedule

# Breast cancer

Let’s start with the first scenario. Imagine the following situation: Ms. Jones has a family history of breast cancer – if it is detected at an early stage breast cancer is often treatable. She could choose to have a genetic test carried out by her doctor to determine whether she has an increased risk of developing breast cancer before the age of 70.

1. What, in your opinion, could motivate Ms. Jones to have such a genetic test, what could deter her from doing so?
2. What kinds of additional information might someone need to decide whether they should have a genetic test carried out in this situation?

Now let’s imagine that Ms. Jones has decided to have the genetic test: The results show that her risk of developing breast cancer before the age of 70 is increased and turns out to be 55-65%. This means that 55-65 people out of every 100 people with this kind of genetic profile would develop the disease.

1. To what extent could the test results influence Ms. Jones’ well-being, her attitudes towards life and her behavior? [How might she feel if she needed to make changes to her lifestyle to try and reduce her risk of developing breast cancer?] [What kinds of additional information might she need to help her deal with this test result?]
2. Should Ms. Jones inform other people about the results of her genetic test? If yes, who (and who not) should she inform, and why? Would you inform other people about the results of this genetic test, if you were in a similar situation?
3. Early-onset Alzheimer’s disease

Now we’ll change the scenario a bit to a different disease: Imagine now that it’s not about the risk of developing breast cancer but about the risk of developing an early-onset form of Alzheimer’s disease. This disease is not yet treatable.

1. How would you feel about someone taking a genetic test for the purpose of predicting their risk of developing Alzheimer’s disease? [Would your previous viewpoint change? To what extent? Why?]

Imagine that Ms. Jones takes this genetic test and the results reveal that her risk of developing Alzheimer’s disease before the age of 65 is at 85%. This means that 85 out of every 100 people with this kind of genetic profile would develop the disease.

1. What are your feelings about this genetic test now? When faced with this kind of test result, would this change your opinion about the value of this kind of genetic test? How?
2. Rheumatoid arthritis

Now we’ll change the scenario again: Imagine now that it’s not about the risk of developing breast cancer or Alzheimer’s disease, but consider the value of a genetic test to determine someone’s risk of developing rheumatoid arthritis. There is currently no cure for this disease, though for many people the symptoms can be managed with long term medication, especially if the disease is diagnosed at an early stage.

1. How would you feel about the value of a genetic test to predict risk of developing rheumatoid arthritis? [Would your previous views change? How? Why?]

Imagine that Ms. Jones takes this genetic test and the results reveal that her risk of developing rheumatoid arthritis before the age of 55 is at 20%. This means that 20 out of every 100 people with this kind of genetic profile would develop the disease.

What are your feelings about this genetic test now?

1. To what extent might this test result influence Ms. Jones’ well-being, or lifestyle? [How do you think she would feel if she were told that she could reduce this risk by a third if she were to give up smoking]
2. What kinds of additional information might she need to deal with this kind of test result
3. Should Ms. Jones inform other people about the results of this genetic test? If yes, who (and who not) should be informed, and why? How is this different, if at all, than your answers for the other diseases?
